# 2017: A Fruitful Year for *Pharmaceuticals*

**DOI:** 10.3390/ph11010001

**Published:** 2017-12-21

**Authors:** Jean Jacques Vanden Eynde

**Affiliations:** Editor-in-Chief, MDPI AG, St. Alban-Anlage 66, Basel CH-4052, Switzerland; Jean-jacques.vandeneynde@ex.umons.ac.be

First of all, let me wish you a healthy and wonderful year in 2018.

## Looking Back on 2017

When we look back on the past year, all of us are proud to announce that 2017 has been a highly fruitful year for our journal. This is wonderful news, rewarding the continuous support offered and confidence expressed by all our authors, readers, reviewers, members of the editorial board, as well as the efforts of our editorial team. 

The most noteworthy fact is, undoubtedly, the release of the CiteScore™ values [1] by Elsevier. Following that new metric, *Pharmaceuticals* obtained a score of 4.90 for 2017, which propels it to #8/168 in the category Pharmaceutical Science and #21/158 in the category Molecular Medicine.

Importantly, also, our journal is now indexed in the Emerging Sources Citation Index (ESCI, Web of Science).

Consequently, it is not surprising that the abstracts of publications in 2017 have been seen more than 400,000 times [2], a record since the inauguration of *Pharmaceuticals*. Incidentally, have you noticed that each of the four issues of Volume 10 (2017) is now illustrated by an elegant colorful cover? [3].

During the past year, twelve distinguished scientists accepted to act as editorial board members, namely:

- Atanas G. Atanasov (University of Vienna, Vienna, Austria);
- Connor R. Caffrey (University of California, San Diego, CA, USA);
- Antoni Camins Espuny (University of Barcelona, Barcelona, Spain);
- Michael Danilenko (Ben-Gurion University of the Negev, Beer Sheva, Israel);
- Christophe Dardonville (Spanish Council for Medicinal Research, Madrid, Spain);
- Gunars Duburs (Latvian Institute of Organic Synthesis, Riga, Latvia);
- Jesus Jimenez-Barbero (Basque Foundation for Science, Bilbao, Spain);
- Carlos Alberto Manssour Fraga (Federal University of Rio de Janeiro, Rio de Janeiro, Brazil);
- Susan Matthews (University of East Anglia, Norwich, UK);
- Mary J. Meegan (Trinity College, Dublin, Ireland);
- Pascal Sonnet (Université de Picardie Jules Vernes, Amiens, France);
- Arpad Szallasi (Anderson Cancer Center, Jacksonville, FL, USA);
- Peter Timmerman (University of Amsterdam, Amsterdam, The Netherlands).

Joachim Jose (Westfälische Wilhelms-Universität, Muenster, Germany) and Klaus Kopka (German Cancer Research Center, Heidelberg, Germany) have joined Annie Mayence (Haute Ecole Provinciale de Hainaut-Condorcet, Belgium) as Associate Editors.

In 2017, we completed six remarkable Special Issues on the following subjects:

- Emerging theranostic tracers in the context of radiopharmaceutical drug development [4], K. Kopka and E. Eppard acted as guest editors;
- Nutraceuticals and botanicals: bioactive molecules and therapeutic properties for human health [5], D. Donno acted as guest editor;
- Selected papers from the 2nd International Electronic Conference on Medicinal Chemistry [6], J.J. Vanden Eynde acted as guest editor;
- Glycosaminoglycans and proteoglycans [7], B. Mulloy acted as guest editor;
- Tissue protective agents: new drugs and technologies [8], S. Collina and S. Rossi acted as guest editors;
- Epilepsy and neurodegeneration: current therapeutic implications [9], A. Camins Espuny acted as guest editor.

Two books have been released: “*An Updated View on an Emerging Target: Selected Papers from the 8th International Conference on Protein Kinase CK2*” [10] (J. Jose, M. Le Borgne, L. A. Pinna, and M. Montenarh, Eds.) and “*Identification and Characterization of Antimicrobial Peptides with Therapeutic Potential*” [11] (G. Wang, Ed.).

After an evaluation of projects by our scientific board, a Travel Award [12] has been offered to Diana I.S.P. Resende, Postdoc fellow at CIIMAR—Interdisciplinary Centre of Marine and Environmental Research, University of Porto (Portugal), whose research focuses on novel marine products with biotechnological applications. She presented the work “Synthetic Strategies for the Synthesis of Quinazoline Alkaloid Derivatives with Promising Biological Activities” at the 20th European Symposium of Organic Chemistry (ESOC) in Cologne (Germany).

*Pharmaceuticals* was again the organizer of the 3rd International Electronic Conference of Medicinal Chemistry [13]. The Conference brought together around 300 authors from 34 different countries (Austria, Belgium, Brazil, Bulgaria, China, Cuba, Ecuador, Egypt, Finland, France, Germany, Greece, India, Indonesia, Ireland, Japan, Latvia, Macedonia, Mexico, Morocco, Nigeria, Pakistan, Poland, Portugal, Romania, Russia, Serbia, South Africa, Spain, Switzerland, Ukraine, United Kingdom, United States of America, and Uruguay). The event hosted more than 70 presentations, keynotes, videos, and posters. Two special round tables enabled participants to focus on nanomedicines, thanks to our media partner Precision Nanosystems, and on parasitic diseases thanks to Dr. C.R. Caffrey from the University of California at San Diego (CA, USA). A brief summary of some of the outstanding works will be presented in a meeting report and a Special Issue to be published soon. The Scientific Advisory Committee elected the work of Soubhye J. (Université Libre de Bruxelles, Belgium), entitled “Dual Anti-Inflammatory and Anti-Bacterial Effects of Phenylhydrazide and Phenylhydrazone Derivatives” as the best presentation [14]. Mention must be made that the virtual exhibition hall attracted 20 media partners to whom we are grateful for having widely publicized the event (AKos, Analis, Bionity, Chemie.de, Cherry Biotech, Elveflow, Eurisotop, Hielscher, Hitgen, ImaBiotech, Latoxan, Linkam, Magritek, Precision Nanosystems, Reaction Biology Corp., Sairem, SimulationPlus, SnapDragon, the journal *Tropical Medicine and Infectious Disease*, and Wylton). 

## Looking Forward to 2018

The topical section “Choices of the Journal” [15], created in 2015, will remain open in 2018, knowing that it enables guest editors as well as editorial board members to invite leading investigators to share their knowledge with our readers.

Maintaining the high quality of our publications and increasing the visibility of our journal remain essential targets on our road map. We are already working in that direction and we are collecting applications for a Travel Award for a PhD student to attend a conference in 2018 [16]. Moreover, we are pleased to announce that the journal will sponsor the Fourth International Electronic Conference on Medicinal Chemistry [17], which will take place online in November 2018. 
